# Circadian and Dopaminergic Regulation of Fatty Acid Oxidation Pathway Genes in Retina and Photoreceptor Cells

**DOI:** 10.1371/journal.pone.0164665

**Published:** 2016-10-11

**Authors:** Patrick Vancura, Tanja Wolloscheck, Kenkichi Baba, Gianluca Tosini, P. Michael Iuvone, Rainer Spessert

**Affiliations:** 1 Institute of Functional and Clinical Anatomy, University Medical Center of the Johannes Gutenberg University, Mainz, Germany; 2 Neuroscience Institute and Department of Pharmacology and Toxicology, Morehouse School of Medicine, Atlanta, Georgia, United States of America; 3 Department of Ophthalmology, Emory University School of Medicine, Atlanta, Georgia, United States of America; Pennsylvania State University, UNITED STATES

## Abstract

The energy metabolism of the retina might comply with daily changes in energy demand and is impaired in diabetic retinopathy—one of the most common causes of blindness in Europe and the USA. The aim of this study was to investigate putative adaptation of energy metabolism in healthy and diabetic retina. Hence expression analysis of metabolic pathway genes was performed using quantitative polymerase chain reaction, semi-quantitative western blot and immunohistochemistry. Transcriptional profiling of key enzymes of energy metabolism identified transcripts of mitochondrial fatty acid *β*-oxidation enzymes, i.e. *carnitine palmitoyltransferase-1α* (*Cpt-1α*) and *medium chain acyl-CoA dehydrogenase* (*Acadm*) to display daily rhythms with peak values during daytime in preparations of the whole retina and microdissected photoreceptors. The cycling of both enzymes persisted in constant darkness, was dampened in mice deficient for dopamine D4 (D_4_) receptors and was altered in db/db mice—a model of diabetic retinopathy. The data of the present study are consistent with circadian clock-dependent and dopaminergic regulation of fatty acid oxidation in retina and its putative disturbance in diabetic retina.

## Introduction

The mammalian retina is a tissue of highest energy demand and oxygen consumption and this situation is even intensified during nighttime when membrane depolarization and the release of neurotransmitter by photoreceptor cells (PRCs) additionally increases energy demands [1, 2]. To comply with these 24-hour changes in metabolic demands functional adaptation of the retina to the environmental lighting conditions might be required.

Daily adaptation of retinal physiology [3, 4] depends on an intrinsic circadian clock system [5, 6] that encompasses various cellular clocks localized in different cell types of the retina [7, 8], including PRCs [9, 10], amacrine cells [11], ganglion cells [12], and Müller glial cells [13]. To fulfill its function the retinal clock system acts through the neuromodulators melatonin and dopamine, which are rhythmically released from PRCs and inner retinal neurons including amacrine cells and interplexiform neurons, respectively [14]. Both neuromodulators play opposing roles in retinal adaptation [15, 16]. Melatonin is mainly produced at the night and contributes to dark adaptation of the retina through G-protein receptors named melatonin type 1 (MT_1_) and type 2 (MT_2_) receptors [17]. On the other hand, dopamine release occurs during the day to promote the adaptation of PRCs to light via dopamine D4 (D_4_) receptors [18, 19].

Similar to other brain areas the energy metabolism of the retina is thought to be glycolytic, i.e. oxidizing glucose rather than fatty acids for fuel. However, the expression of fatty acid oxidation (FAO) enzymes in the retina [20, 21] and the progressive retinopathy that occurs with inherited deficiencies of some of them [22, 23] suggest that β-oxidation is also important for retinal physiology, even though the specific role of FAO in retinal metabolism is unknown and the transport of fatty acids across the blood-brain barrier is limited.

The major regulatory enzyme of FAO is the mitochondrial outer membrane protein carnitine palmitoyltransferase-1 (Cpt-1), which facilitates the transport of long chain fatty acids into the mitochondrial matrix. Cpt-1 occurs in two catalytic active isoforms that are Cpt-1α (liver isoform), which is present in all tissues except skeletal muscle and adipose tissue, and Cpt-1β (skeletal muscle isoform), which is expressed in several tissues including the skeletal muscle, adipose tissue, heart and brain [24]. A third isoform, the brain-specific enzyme Cpt-1c [25], does not catalyze the prototypical reaction but appears to be involved in the regulation of energy homeostasis by controlling food intake [26].

Each of the four reactions in the β-oxidation pathway in the strict sense (dehydrogenation, hydration, oxidation to form a β-keto acid, and thiolysis) is catalyzed by several different enzymes that have specificity for substrates of different chain length. For example, four proteins catalyze the first dehydrogenation step, i.e. very-long-chain, long-chain, medium-chain and short-chain acyl-CoA dehydrogenases (Acadvl, Acadl, Acadm, Acads). A feature unique to the long-chain FAO is that the last three steps do not occur in the mitochondrial matrix but in a membrane-bound complex harboring the three relevant enzyme activities called trifunctional protein (TP). The trifunctional protein consists of four alpha subunits and four beta subunits, the alpha subunits containing enoyl-CoA hydratase and 3-hydroxyacyl-CoA dehydrogenase activities and the beta subunits performing the beta-ketothiolase activity. These subunits are encoded by the *Hadha* (OMIM # 600890) and *Hadhb* (OMIM # 143450) genes, respectively [27].

In peripheral tissues such as liver, one of the key processes regulated by circadian clock systems is energy metabolism including FAO [28] and loss or disruption of clock control is associated with abnormal metabolic phenotypes that exhibit symptoms of metabolic syndrome and type II diabetes [29]. To gain insight whether clock-dependent regulation of energy metabolism and its impairment in type II diabetes is also evident in the retina, a tissue of the CNS that has to comply with daily changes in energy demand was the aim of the present study.

## Material and Methods

### Animals

Adult (age of 10–12 weeks) male and female mice with intact PRCs not carrying the *rd* mutation were used in this study. With the exception of the mouse model for diabetic retinopathy (C57BL/6Jb db/+, C57BL/6Jb db/db), the mice used were melatonin-proficient (C3H/He, C3H/f^+/+^MT1^+/+^, C3H/f^+/+^MT1^-/-^, C3H/f^+/+^Drd4^+/+^ and C3H/f^+/+^Drd4^-/-^). When indicated mice deficient for melatonin receptor type 1 (*Mt*_*1*_^*-/-*^) or dopamine D4 receptors (*Drd*_*4*_^*-/-*^) were used, genotyped by PCR analysis of genomic DNA. Diabetic (db/db) and non-diabetic (db/+) mice were purchased from Jackson Laboratory (Bar Harbor, ME, USA). They were checked for body-weight and blood glucose level by tail vein sampling using Accu-Check Aviva reagent strips (Roche Diagnostics, Mannheim, Germany) at the age of 10 weeks. Diabetic mice displayed enhanced values of blood glucose (397 ± 14 mg/dl) and bodyweight (46 ± 3 g) as compared to non-diabetic mice (blood glucose level: 138 ± 4 mg/dl; bodyweight: 25 ± 1 g). Mice were kept under light/dark 12:12 (LD) for 3 weeks under standard laboratory conditions (illumination with 200 lux at cage level during the day and dim red light (<5 lux) during the night, 20 ± 1°C, water and food ad libitum) and sacrificed at 3-h intervals (two animals / four retinae per time-point) over a period of 24 hours by decapitation following anesthesia with carbon dioxide. In order to determine putative circadian expression of genes, mice previously adapted to LD were housed in constant darkness (DD) for one cycle and sacrificed during the next cycle. Animal experimentation was carried out in accordance with the National Institutes of Health Guide on the Care and Use of Laboratory Animals and the ARVO Statement for the Use of Animals in Ophthalmic Vision Research, and approved by the Institutional Animal Care and Use Committees of Morehouse School of Medicine, Emory University, and the European Communities Council Directive (86/609/EEC).

### Retina sampling

Eyes of mice were enucleated and incised on the corneal limbus. After discarding the lens and vitreous, the retinas were rapidly collected, pooled and immediately frozen or processed for laser microdissection and pressure catapulting (LMPC). All dissections during the dark phase were done under dim red light. In order to prepare the retinas for LMPC, the HOPE technique [30] was applied for fixation. In this procedure fresh retinas were fixed in HOPE I (DCS, Hamburg, Germany), at 0–4°C for 48 h. Subsequently, dehydration of the retinas was performed with a mixture of HOPE II solution (DCS, Hamburg, Germany) and acetone for 2 h at 0–4°C, followed by dehydration in pure acetone at 0–4°C (repeated twice). Tissues were then embedded with low-melting point paraffin (T_m_ 52–54°C) and sectioned (10 μm) on membrane-mounted slides (DNase/RNase free PALM MembraneSlides, P.A.L.M., Bernried, Germany). Subsequently sections were de-paraffinized with isopropanol (2 × 10 min each at 60°C), stained using cresyl violet (1% w/v cresyl violet acetate in 100% ethanol), briefly washed in 70% and 100% ethanol respectively, and then air-dried.

### Isolation of photoreceptor cells

To isolate PRCs (rod and cones) from the stained sections in a contact and contamination-free manner, LMPC was performed with a PALM MicroBeam system (Zeiss MicroImaging, Munich, Germany) running PALM RoboSoftware (P.A.L.M., Bernried, Germany) as described previously [10]. In brief, these cells were selected, cut and catapulted into the caps of 0.5 ml microfuge tubes with an adhesive filling (PALM AdhesiveCaps, P.A.L.M., Bernried, Germany) by utilizing a pulsed UV-A nitrogen laser under the 10× objective. To reach total average sample sizes of 4 million square microns per tube, smaller areas of the sections were pooled. The purity grades of the preparations obtained were verified by using specific gene markers of PRCs, namely *rhodopsin* (*Rho*) and *neural retina leucine zipper* (*Nrl*) as markers for rods [31, 32] as well as of inner retinal neurons, namely *tyrosine hydroxylase* (*Th*) as a marker for amacrine cells [33] and *metabotropic glutamate receptor 6* (*mGluR6*) as a marker of on-bipolar cells [34].

### RNA extraction, reverse transcription (RT) and quantitative polymerase chain reaction (qPCR)

Using the RNeasy Micro Kit (Qiagen, Hilden, Germany) RNA was extracted from the tissue samples as described [35]. The amount of extracted RNA was determined by measuring the optical density at 260 and 280 nm. Subsequently first stranded cDNA was synthesized by using the Verso cDNA Kit (Abgene, Hamburg, Germany), following the manufacturer’s instructions. Briefly, 4 μl RNA solution was reverse transcribed using anchored oligo-dT primers in a final volume of 20 μl. Following dilution of the obtained cDNA sample in RNase-free water (1:4) quantitative PCR, with aliquots of 5 μl being used, was performed as described [36]. PCR amplification and quantification was carried out in duplicates using an i-Cycler (BioRad, Munich, Germany) according to the following protocol: denaturation for 30 seconds at 95°C, followed by 45 cycles of 5 seconds at 95°C and 30 seconds at 60°C. By using agarose gel electrophoresis, the generated amplicons for all genes under examination were shown to possess the predicted sizes (Table 1). The amount of mRNA in the samples was calculated from the measured threshold cycles (C_t_) using an internal standard curve with 10-fold serial dilutions (10^1^−10^8^ copies/μl). Expression levels of each transcript were normalized by comparison in parallel with the amount of both *Gapdh* mRNA and *18S* rRNA present.

**Table 1:** Primer sequences used for qPCR.

Gene	Accession Number	Primer Sequence 5′ to 3′	Length of PCR Product [bp]
*18S*	NR_003278.3	Forward: CAACACGGGAAACCTCAC; Reverse: TCGCTCCACCAACTAAGAAC	110
*Acadl*	NM_007381.4	Forward: GTGCCATAGCCATGACAGAG; Reverse: CACGACGATCACGAGATCAC	146
*Acadm*	NM_007382.5	Forward: TACCCGTTCCCTCTCATC; Reverse: CCCATACGCCAACTCTTC	129
*Acads*	NM_007383.3	Forward: CTTGGCTGCCTCTTTACC; Reverse: GTCCTGTCCCTTGTGTTC	133
*Acadvl*	NM_017366.3	Forward: GCTCGGATGGCTATTCTG; Reverse: GATGGCGGCTTCTATCTG	102
*Cpt-1a*	NM_013495.2	Forward: GCCATCTGTGGGAGTATGTC; Reverse: TGTAGCCTGGTGGGTTTG	112
*Cpt-1b*	NM_009948.2	Forward: CTTTCACCTGGGCTACAC; Reverse: CCTTGGCTACTTGGTACG	137
*Cpt-1c*	NM_153679.2	Forward: CCGGATTACGTTTCCTCTG; Reverse: CGGTGAGAGTCTGTTTCTG	140
*Cpt-2*	NM_009949.2	Forward: TACCAGCGGATAAACCAC; Reverse: CAATGCCAAAGCCATCAG	103
*Dbp*	NM_016974.3	Forward: GGAGGTGCTAATGACCTTTG; Reverse: GGACTTTCCTTGCCTTCTTC	146
*G6p*	NM_008061.4	Forward: CTCACACCACCTTCTCTATC; Reverse: AACAGTTGCCTACCAGAC	125
*Gapdh*	BC082592	Forward: CATCCCAGAGCTGAAC; Reverse: TCAGATGCCTGCTTCAC	144
*Gsk3b*	NM_019827.6	Forward: TTCACCCTTCCCACTCTG; Reverse: TGCACCTCCTGTCTACAC	145
*Gys1*	NM_030678.3	Forward: GAAGCCCTGGGATCTAAC; Reverse: GGCCTTGAACTCCCTATG	139
*Hadha*	NM_178878.2	Forward: GTCTTTGGGCTTGGCTTTC; Reverse: ATAGGCAGACTCGTACTTCC	109
*Hadhb*	NM_001289798.1	Forward: GATCACCTCCTCTGGAGAAG; Reverse: CACAGGCAGCCACTAAAG	147
*Hk1*	NM_010438.3	Forward: CGTCCGTAACATCCTGATTG; Reverse: AATCGGTCACTCTCGATCTG	126
*mGluR6*	NM_173372.2	Forward: CAAGACCAACCGCATCTAC; Reverse: ACGCTATCACTCCCACTAC	134
*Nrl*	NM_008736.3	Forward: GTGGAGGAACGGTCCAGATG; Reverse: GAACTGGAGGGCTGGGTTAC	149
*Pck2*	NM_028994.2	Forward: CAAAGCCAGCTAGACAAC; Reverse: ATAGGGTCCTTCCAAGTG	144
*Rho*	NM_145383.1	Forward: CTGAGGGCATGCAATGTTCA; Reverse: CATAGCAGAAGAAGATGACG	132
*Th*	NM_009377.1	Forward: CAGCCCTACCAAGATCAAAC; Reverse: GTACGGGTCAAACTTCACAG	129

### Western blot analysis

For Western blot analysis, samples were loaded on 4–12% NuPAGE Novex Bis-Tris gels (Invitrogen, Carlsbad, CA, USA), separated and then blotted onto PVDF membrane (Westran S, Whatman Inc., Sanford, ME, USA). For immunodetection, membranes were blocked in 5% skimmed milk powder and incubated with anti-Cpt-1α monoconal antibody (1:500; Proteintech, Rosemont, IL, USA; 66039-1-lg) overnight at 4°C. Using an ECL detection system (GE Healthcare Amersham, Freiburg, Germany) the horseradish-peroxidase-conjugated secondary antibodies (goat anti-mouse-HRP 1:5000; Sigma-Aldrich, St. Louis, MO, USA; A0545) were visualized. Rabbit anti-β-actin polyclonal antibody (1:300; Sigma-Aldrich, St. Louis, MO, USA; A2066) was used to control for equal protein loading. Densitometry measurement was performed using Image Lab 4.1 (Bio-Rad Laboratories, Hercules, CA, USA).

### Immunohistochemistry

Eyes were embedded in optimal cutting temperature (OCT) compound (Tissue-Tek; Sakura Finetek, Tokyo, Japan) and frozen in a tank filled with melting 2-methyl-butane (VWR, Radnor, PA, USA) placed in liquid nitrogen. Cryosections (10μm) were incubated with 0.1% Tween 20 in PBS. After washing with PBS, sections were treated with blocking solution (0.5% cold-water fish gelatin plus 0.1% ovalbumin in PBS) for 30 minutes, and then incubated with primary antibody (anti-Cpt-1α monoclonal antibody, Proteintech, Rosemont, IL, USA, 66039-1-lg; anti-Centrin3 polyclonal antibody, kindly provided by Prof. Wolfrum, Institute of Zoology, Johannes Gutenberg University, Mainz, Germany) in blocking solution overnight at 4°C. Following removal of the primary antibody, slides were washed and subsequently incubated with Alexa Fluor488 or Alexa Fluor568 conjugated donkey anti-mouse or donkey anti-rabbit secondary antibodies (Molecular Probes, Leiden, The Netherlands) for 1 hour in blocking solution at room temperature. Cell nuclei were counterstained with DAPI (Thermo Fisher Scientific, Waltham, USA). Negative immunohistochemistry controls were performed in parallel by omission of primary antibody. After they were washed, sections were mounted in Mowiol 4.88 (Hoechst, Frankfurt, Germany). Mounted retinal sections were examined by DMRP microscope (Leica, Bensheim, Germany) and images were obtained with an ORCA ER charge-coupled device camera (Hamamatsu, Herrsching, Germany).

### Statistical analysis

All data are expressed as the mean ± standard error of the mean (SEM) of four independent (qPCR) and eight (Western blot) experiments. Transcript levels were calculated relative to average expression of each dataset throughout 24 hours to plot temporal expression. Cosinor analysis was used to evaluate variations among the groups in the 24-h profile and to fit sine-wave curves to the circadian data to mathematically estimate the time of peaking gene expression (acrophase) and to assess the amplitude [37, 38]. The model can be expressed according to the equation: f(t) = A + B cos [2π (t + C) / T] with the f(t) indicating relative expression levels of target genes, t specifying the time of sampling (h), A representing the mean value of the cosine curve (mesor; midline estimating statistic of rhythm), B indicating the amplitude of the curve (half of the sinusoid) and C indicating the acrophase (point of time, when the function f(t) is maximum). T gives the time of the period, which was fixed at 24 hours for this experimental setting. Protein levels were calculated relative to maximal expression of each dataset throughout 24 hours to plot temporal expression. One-way ANOVA (one way analysis of variance) was used to evaluate variations among the groups in the 24-h profile. Significance of daily regulation was defined by showing a p < 0.05.

## Results

### Screening of key enzymes of metabolism to be under daily and circadian regulation in retina

**Fig 1:**
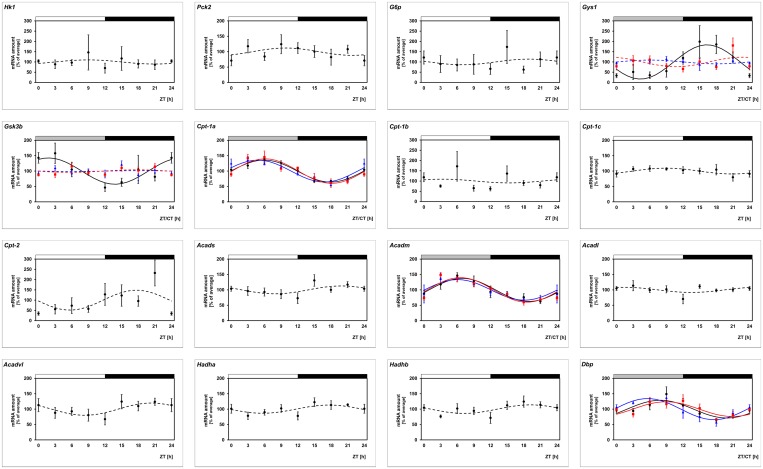
Transcriptional screening of key enzymes of energy metabolism to be under daily and circadian regulation in retina and photoreceptor cells. Transcript levels of key enzymes of glycolysis (*Hk1*), gluconeogenesis (*Pck2* and *G6p*), glycogen synthesis (*Gys1* and *Gsk3*) and fatty acid oxidation (*Cpt-1a*, *Cpt-1b*, *Cpt-1c*, *Cpt-2*, *Acadm*, *Acads*, *Acadl*, *Acadvl*, *Hadha*, *Hadhb*) are recorded in preparations of the whole retina under light/dark (LD) 12:12 (black lines), in preparations of the whole retina under constant darkness (DD) (blue lines) and in microdissected photoreceptor cells (PRCs) under light/dark (LD) 12:12 (red lines) using qPCR. The mRNA levels are plotted as a function of Zeitgeber time (ZT) and the lines represent the periodic sinusoidal functions determined by cosinor analysis (solid and broken line for p < 0.05 and p > 0.05 in cosinor analysis). Data represent a percentage of the average value of the transcript amount during the 24-h period. Statistical analysis of transcriptional profiling is provided in Table 2. The value of ZT0 was plotted twice at both ZT0 and ZT24. The solid bars indicate the dark period. Each value represents mean ± SEM (n = 4; each n represents two animals / four retinae). Note that the FAO genes *Cpt-1α* and *Acadm* as well as the reference gene *Dbp* exhibit significant fluctuations in all applied settings.

To gain insight of daily regulation of energy metabolism in the retina, transcriptional profiling of metabolic key enzymes was performed (Fig 1, black lines; for statistical analysis, see Table 2). This approach determines the transcripts of key enzymes of FAO (*Cpt-1α*, *Acadm*) and glycogen synthesis (*Gys1*, *Gsk3b*) to be under daily regulation in the retina.

**Table 2:** Statistical analysis of transcriptional profiling illustrated in Fig 1.

Gene	Retina (C3H/He (rd^++^)); LD				Retina (C3H/He (rd^++^)); DD				PRCs (C3H/He (rd^++^)); LD			
	1-way ANOVA	Cosinor Analysis			1-way ANOVA	Cosinor Analysis			1-way ANOVA	Cosinor Analysis		
	p-Value	p-Value	Acrophase (h)	Amplitude (%)	p-Value	p-Value	Acrophase (h)	Amplitude (%)	p-Value	p-Value	Acrophase (h)	Amplitude (%)
*Acadl*	= 0.144	> 0.05	-	-	-	-	-	-	-	-	-	-
*Acadm*	< 0.001	< 0.05	7.31	37.68	= 0.006	< 0.05	5.72	33.53	< 0.001	< 0.05	7.05	39.97
*Acads*	= 0.169	> 0.05	-	-	-	-	-	-	-	-	-	-
*Acadvl*	= 0.333	> 0.05	-	-	-	-	-	-	-	-	-	-
*Cpt-1α*	< 0.001	< 0.05	5.95	35.87	< 0.001	< 0.05	4.19	35.74	< 0.001	< 0.05	6.71	39.92
*Cpt-1β*	= 0.194	> 0.05	-	-	-	-	-	-	-	-	-	-
*Cpt-1γ*	= 0.547	> 0.05	-	-	-	-	-	-	-	-	-	-
*Cpt-2*	= 0.484	> 0.05	-	-	-	-	-	-	-	-	-	-
*Dbp*	= 0.020	< 0.05	7.94	28.91	= 0.001	< 0.05	4.99	34.42	= 0.016	< 0.05	8.63	23.95
*G6p*	= 0.671	> 0.05	-	-	-	-	-	-	-	-	-	-
*Gsk3b*	= 0.048	< 0.05	5.00	42.68	= 0.489	> 0.05	-	-	= 0.159	> 0.05	-	-
*Gys1*	= 0.025	< 0.05	15.02	83.45	= 0.767	> 0.05	-	-	= 0.015	> 0.05	-	-
*Hadha*	= 0.160	> 0.05	-	-	-	-	-	-	-	-	-	-
*Hadhb*	= 0.196	> 0.05	-	-	-	-	-	-	-	-	-	-
*Hk1*	= 0.929	> 0.05	-	-	-	-	-	-	-	-	-	-
*Pck2*	= 0.556	> 0.05	-	-	-	-	-	-	-	-	-	-

Daily regulation of a gene may depend on a circadian clock or light/dark-transitions. To identify genes under circadian regulation, 24-h profiling of genes shown to be rhythmic under LD conditions (*Cpt-1α*, *Acadm*, *Gys1*, *Gsk3b*) was performed in mice adapted to DD (Fig 1, blue lines; for statistical analysis, see Table 2). Under these conditions 24-h regulation was maintained for the enzymes of FAO (*Cpt-1α*, *Acadm*) but not for those of glycogen synthesis (*Gys1*, *Gsk3b*). These observations are consistent with circadian regulation of genes of the FAO pathway and non-circadian regulation of genes of the carbohydrate metabolism.

### Localization and daily regulation of Cpt-1α protein

**Fig 2:**
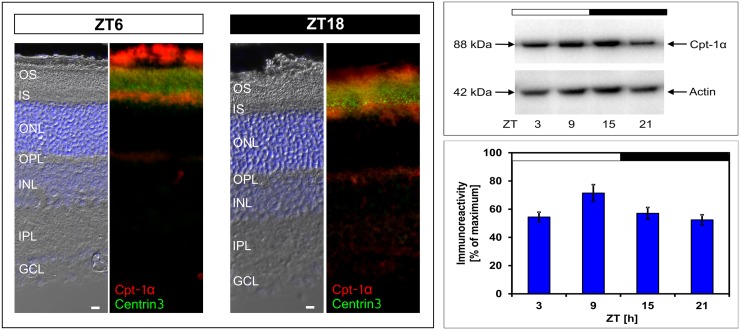
Localization and 24-h profiling of Cpt-1α protein in retina. Left column: Micrographs of coronal sections of the retina, labelled for Cpt-1α (red) and Centrin3 (green) at Zeitgeber times (ZT) 6 and 18. The representative immunofluorescent images show that Cpt-1α protein is primarily located in the inner segments of PRCs with no difference in the localization between ZT6 and ZT18. The upper right column shows a representative Western blot with Cpt-1α immunostaining at about 88 kDa and β-Actin staining as a loading control. The diagram on the lower right column displays the quantification of Cpt-1α immunoreactivity in relation to the corresponding β-actin signal to which it was normalized. Data were obtained using densitometric measurement and represent percentages of the overall maximal value. Each value represents mean ± SEM (n = 8; each n represents one animal / two retinae). The solid bars indicate the dark period. Note that Cpt-1α immunoreactivity exhibits daily changes with peak expression around ZT9 (*p < 0.05 in one-way ANOVA). OS, outer segment; IS, inner segment; ONL, outer nuclear layer; OPL, outer plexiform layer; INL, inner nuclear layer; IPL, inner plexiform layer; GCL, ganglion cell layer. Scale bar, 10 μm.

To investigate circadian regulation of FAO, we focused on *Cpt-1α*, which encodes the major regulatory enzyme of FAO. In the first step the localization of Cpt-1α protein was investigated by means of fluorescence microscopy (Fig 2, left column) using an antibody that recognizes a band of ~ 88 kDa, a molecular mass in the range of the molecular mass predicted from the *Cpt-1α* gene (Fig 2, right column). Immunohistochemistry was performed using double labeling analysis for Cpt-1α and Centrin3, a marker of the connecting cilium of PRCs [39]. Irrespective of the ZT, Cpt-1α-immunoreactivity occurred in PRCs where it co-localized with Centrin3.

In the context of a putative role of *Cpt-1α* in circadian regulation of FAO and energy supply, the question of whether 24-h changes in *Cpt-1α* transcription results in corresponding variations in Cpt-1α protein was addressed using Western blot analysis (Fig 2, right column). The intensity of Cpt-1α immunostaining displayed daily changes with elevated value around ZT9. This suggests that 24-h changes in *Cpt-1α* mRNA result in corresponding changes in Cpt-1α protein amount. However, the daytime-increase in *Cpt-1α* expression was at the protein level (approximately 25%) weaker than at the transcript level (approximately 50%). This may reflect that for a given LD cycle Cpt-1α de-novo formation only partly accounts for the entire amount of Cpt-1α protein present in the retina.

### Daily regulation of *Cpt-1α* mRNA in photoreceptor cells

Localization of Cpt-1α suggests that its daily regulation occurs in the PRC. To test this assumption daily profiling of *Cpt-1α* was performed in PRCs enriched using the LMPC technique. In this approach the purity grades of the PRC preparations used were verified by using specific gene markers of PRCs, namely *rhodopsin* (*Rho*) and *neural retina leucine zipper* (*Nrl*) as markers for rods [31, 32] as well as of inner retinal neurons, namely *tyrosine hydroxylase* (*Th*) as a marker for amacrine cells [33] and *metabotropic glutamate receptor 6* (*mGluR6*) as a marker of on-bipolar cells [34]. In comparison to whole retina preparations, in PRCs collected with LMPC, the ratio of *Nrl* to *Th* and *mGluR6* was increased 84-fold and 5-fold, respectively and that of *Rho* to *Th* and *mGluR6* was increased 11-fold and 2-fold, respectively. It was seen that the transcript level of *Cpt-1α* exhibits a daily rhythm in PRCs (Fig 1, red lines; for statistical analysis, see Table 2) with a profile resembling that obtained from preparations of the whole retina (Fig 1, black lines; for statistical analysis, see Table 2). Therefore, daily changes in *Cpt-1α* expression observed in preparations of the whole retina may mainly derive from PRCs.

### Daily regulation of *Cpt-1α* fails in dopamine D4 receptor deficient mice

**Fig 3:**
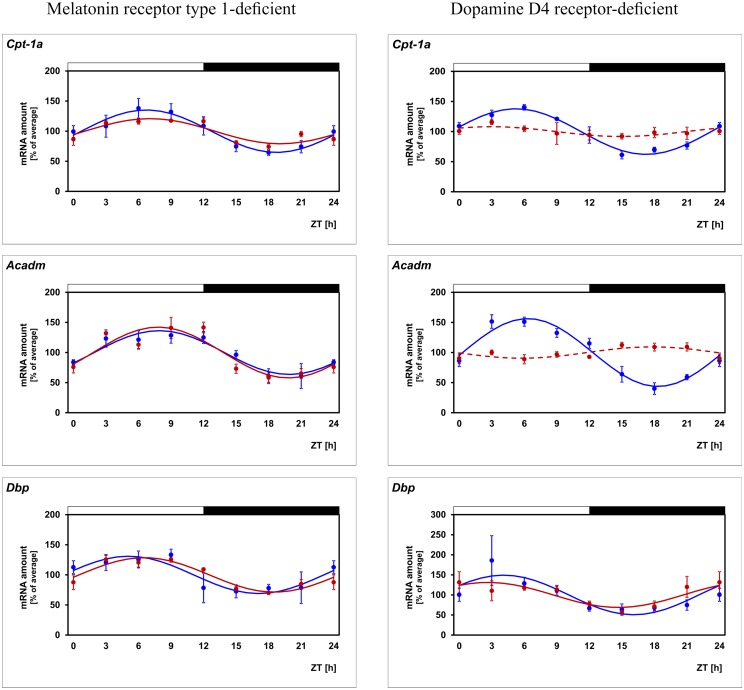
Daily profiling of enzymes of the FAO pathway in mice deficient for either melatonin receptor type 1 or dopamine D4 receptor. Transcript levels of *Cpt-1α*, *Acadm* and *Dbp* are recorded in WT mice (blue lines) and in mice deficient for either melatonin receptor type 1 (left column, red lines) or dopamine D4 receptor (right column, red lines) in preparations of the whole retina under light/dark (LD) 12:12 determined by using qPCR. The mRNA levels are plotted as a function of Zeitgeber time (ZT). The lines represent the periodic sinusoidal functions determined by cosinor analysis (solid and broken line for p < 0.05 and p > 0.05). Data represent a percentage of the average value of the transcript amount during the 24-h period. Statistical analysis of transcriptional profiling is provided in Table 3. The value of ZT0 was plotted twice at both ZT0 and ZT24. The solid bars indicate the dark period. Each value represents mean ± SEM (n = 4; each n represents two animals / four retinae). Note that periodicity of *Cpt-1α* and *Acadm*, but not that of the reference gene *Dbp*, is depressed in mice deficient for dopamine D4 receptors.

In order to evaluate the contribution of melatonin and dopamine to daily regulation of *Cpt-1α*, 24-h profiling of the gene was performed in mice deficient for MT_1_ or D_4_ receptor (Fig 3; for statistical analysis, see Table 3). The daily rhythm of *Cpt-1α* was seen to persist in *Mt*_*1*_ deficient mice even though with reduced amplitude. This observation suggests that daily regulation of *Cpt-1α* does not require but might be reinforced by the pulsatile melatonin signal. More important, daily regulation of *Cpt-1α* failed in *Drd*_*4*_ deficient mice. This suggests that daily regulation of the gene requires dopamine signaling via D_4_ receptors. Since dopamine release and D_4_ receptor stimulation occurs in a circadian manner this finding is consistent with a hypothetical concept in which *Cpt-1α* expression depends on a clock-driven dopamine signal.

**Table 3:** Statistical analysis of transcriptional profiling illustrated in Fig 3.

**Gene**	***C3H/f*^*+/+*^*Mt1*^*+/+*^**				***C3H/f*^*+/+*^*Mt1*^*-/-*^**			
	**1-way ANOVA**	**Cosinor Analysis**			**1-way ANOVA**	**Cosinor Analysis**		
	**p-Value**	**p-Value**	**Acrophase (h)**	**Amplitude (%)**	**p-Value**	**p-Value**	**Acrophase (h)**	**Amplitude (%)**
*Acadm*	= 0.001	< 0.05	9.00	36.32	< 0.001	< 0.05	7.99	42.22
*Cpt-1α*	= 0.002	< 0.05	7.98	35.23	< 0.001	< 0.05	7.24	20.90
*Dbp*	= 0.040	< 0.05	6.73	30.95	< 0.001	< 0.05	6.74	28.63
**Gene**	***C3H/f***^***+/+***^***Drd***_***4***_^***+/+***^				***C3H/f***^***+/+***^***Drd4***^***-/-***^			
	**1-way ANOVA**	**Cosinor Analysis**			**1-way ANOVA**	**Cosinor Analysis**		
	**p-Value**	**p-Value**	**Acrophase (h)**	**Amplitude (%)**	**p-Value**	**p-Value**	**Acrophase (h)**	**Amplitude (%)**
*Acadm*	< 0.001	< 0.05	6.93	56.43	= 0.318	> 0.05	-	-
*Cpt-1α*	< 0.001	< 0.05	5.66	37.90	= 0.694	> 0.05	-	-
*Dbp*	= 0.016	< 0.05	4.26	49.30	= 0.043	< 0.05	2.60	31.19

### The daily profile of *Cpt-1α* is altered in diabetic mice

**Fig 4:**
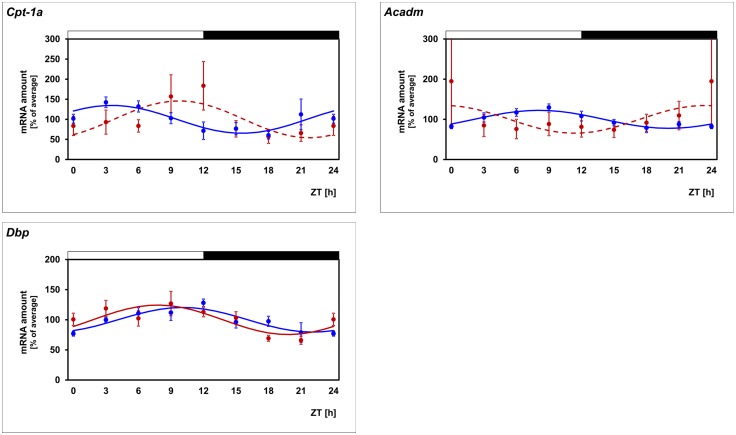
Daily profiling of enzymes of the FAO pathway in db/db mice. Transcript levels of *Cpt-1α* and *Acadm* are recorded in non-diabetic db/+ mice (blue lines) and diabetic db/db mice (red lines) in preparations of the whole retina under light/dark (LD) 12:12 using qPCR. The mRNA levels are plotted as a function of Zeitgeber time (ZT) and the lines represent the periodic sinusoidal functions determined by cosinor analysis (solid and broken line for p < 0.05 and p > 0.05). Data represent a percentage of the average value of the transcript amount during the 24-h period. Statistical analysis of transcriptional profiling is provided in Table 4. The value of ZT0 was plotted twice at both ZT0 and ZT24. The solid bars indicate the dark period. Each value represents mean ± SEM (n = 4; each n represents two animals / four retinae). Note that the daily profiles of *Cpt-1α* and *Acadm* are modulated in db/db mice whereas the reference gene *Dbp* is unaffected.

The transcriptional control of *Cpt-1α* expression in the liver is impaired in type II diabetes mellitus and this contributes to metabolic disorder of the body associated with this disease [40, 41]. For this reason and because dysregulation of the photoreceptor energy metabolism contributes to the pathogenesis of diabetic retinopathy [42] it might also be disturbed in diabetic retinopathy. To test this assumption the db/db mice, a model of Type II diabetes with retinopathy [43], was used. The daily profile in *Cpt-1α* mRNA amount is altered in diabetic (db/db) mice as compared with the non-diabetic phenotype (db/+) (Fig 4; for statistical analysis, see Table 4). Thus in diabetic mice the 24-h rhythm in *Cpt-1α* mRNA tended to persist (even if rhythmicity falls below the significance level in cosinor analysis) but occurred with different phasing. This suggests that daily regulation of FAO is disrupted in diabetic retinopathy.

**Table 4:** Statistical analysis of transcriptional profiling illustrated in Fig 4.

Gene	*db/+ (C57BL/6Jb)*				*db/db (C57BL/6Jb)*			
	1-way ANOVA	Cosinor Analysis			1-way ANOVA	Cosinor Analysis		
	p-Value	p-Value	Acrophase (h)	Amplitude (%)	p-Value	p-Value	Acrophase (h)	Amplitude (%)
*Acadm*	= 0.009	< 0.05	8.77	22.43	= 0.610	> 0.05	-	-
*Cpt-1α*	= 0.007	< 0.05	7.00	18.32	= 0.146	> 0.05	-	-
*Dbp*	= 0.019	< 0.05	11.16	20.44	= 0.010	< 0.05	8.22	24.46

### Daily regulation of *Acadm* mimics that of *Cpt-1α*

Cpt-1α facilitates the transport of long-chain but not medium-chain fatty acids into mitochondrial matrix. Therefore, circadian regulation of another component of the FAO may account for circadian regulation of medium-chain FAO. A candidate is *Acadm*, a gene which encodes the enzyme that catalyzes the first dehydrogenation step of FAO of exclusively medium-chain fatty acids. Remarkably, circadian regulation of *Acadm* was similar to that of *Cpt-1α* (Fig 1; for statistical analysis, see Table 2). This is evident from the observations that circadian *Acadm* regulation was (1) evident in PRCs (Fig 1; for statistical analysis, see Table 2), (2) not detectably altered in *Mt*_*1*_ deficient retinae (Fig 3; for statistical analysis, see Table 3), (3) depressed in *Drd4* deficient retinae (Fig 3; for statistical analysis, see Table 3), and (4) altered in diabetic (db/db) mice (Fig 4; for statistical analysis, see Table 4). Irrespective of lighting conditions and genotype, the profile of *Acadm* expression resembles that of *Cpt-1α* but was phase-delayed relative to it (Figs 1–4; for statistical analysis, see Tables 2–4).

### Daily profiling of the reference gene *Dbp* confirms the validity of the experimental system

To test the validity of the experimental system used and the results obtained, the clock-controlled gene *D site albumin promoter binding protein* (*Dbp*)—whose transcription is directly regulated through the binding of the Clock/Bmal1 complex to E-box motifs in the promoter region of the gene [44]—was monitored in the same transcriptomes as those utilized for *Cpt-1α* and *Acadm* mRNA determination. Consistent with a previous study [45], *Dbp* was observed to be rhythmic under LD conditions (Fig 1, black line; for statistical analysis, see Table 2), under DD conditions (Fig 1, blue line; for statistical analysis, see Table 2), in PRCs (Fig 1, red line; for statistical analysis, see Table 2) and in retina deficient for *Mt*_*1*_ or *Drd*_*4*_ (Fig 3; for statistical analysis, see Table 3). Beyond what was known, *Dbp* rhythmicity was found to be unaffected in diabetic mice in this study (Fig 4; for statistical analysis, see Table 4).

## Discussion

The energy demand of retina and PRCs displays marked daily changes. This requires corresponding changes in energy supply. The findings of the present study suggest that clock-dependent regulation of the energy metabolism contributes to the fulfillment of this challenge and that the FAO pathway—an important source of cellular energy supply through the tricarboxylic acid cycle—plays a role in that matter. This becomes evident from the observation that the gene *Cpt-1α*—encoding the rate-limiting enzyme in the FAO pathway—is under circadian regulation in retina and PRCs. *Cpt-1α* expression peaks during day but energy demand of the retina is increased at dusk [2]. The temporal gap between both parameters is consistent with the principal function of a circadian clock, i.e. to anticipate periodic changes in the environment. Thus clock-dependent up-regulation of *Cpt-1α* during daytime may contribute to provide sufficient energy for the retina at nightfall, taken into account the time required for FAO and subsequent ATP synthesis.

Nutrition of the retina involves FOA [46] but is believed to primarily depend on the supply of blood glucose [47] whose level displays a circadian rhythm with elevated values during the daytime [48]. Therefore, the requirement of changing energy supply of the retina could be achieved by combining clock-dependent regulation of FOA within and clock-dependent regulation of blood glucose outside the retina. Consistent with this assumption the daily change in Cpt-1α protein of approximately 25% (this study) is not likely to fully cover the daily change in energy demand of approximately 75% [2]. Interestingly, expression of *Gys1*—a gene encoding the key enzyme of glycogen synthesis—was seen in the present study to be up-regulated during the second half of the day when glucose supply is high. This might facilitate retinal storage of glucose in terms of glycogen [49] during this time period to address the rising energy demand at nightfall. If this assumption is valid, then the nocturnal increase in the expression of *Gsk3b*—a gene coding for a down-regulator of Gys1 activity—may terminate glycogen formation at dawn when energy demand of the retina attenuates.

Circadian regulation of *Cpt-1α* has previously been reported in liver [50, 51], skeletal muscle [52], and other tissues such as lung, kidney and adrenal gland (CircaDB, Circadian Expression Profiles Data Base; http://circadb.hogeneschlab.org/). The present gene profiling study suggests that circadian regulation of *Cpt-1α* is not only present in peripheral tissue but also in the retina and thus in an area of the brain/CNS. This suggests that circadian control of *Cpt-1α* expression appears to provide a conserved mechanism among distinct tissues to comply with changing energy demands. In tissues other than retina, regulation of *Cpt-1α* expression involves the transcriptional coactivator *Pgc-1α* [53], a key regulator of energy metabolism [54], and the nuclear orphan receptor *Nr4a1* [55], both of which are supposed to mediate circadian regulation of gene transcription in the murine retina [56]. Therefore, *Pgc-1α* and *Nr4a1* are candidates for linking retinal clocks to *Cpt-1α* transcription. Cpt-1α immunostaining is mainly abundant in PRCs (Fig 2). As expected for a mitochondrial membrane-bound enzyme, it localizes primarily to the inner segment, the part of the PRC with the highest density of mitochondria. In the present study circadian regulation of *Cpt-1α* was found to occur in PRCs. For this reason and because PCRs bear D_4_ receptors that are known to address photoreceptor physiology [57, 58], circadian/dopaminergic control of *Cpt-1α* appears to involve the action of the inner retinal dopamine signal on photoreceptor D_4_ receptors.

Circadian regulation of *Cpt-1α* appears to depend on dopamine signaling/D_4_ receptors. This is evident from the present finding that daily periodicity of *Cpt-1α* mRNA is prevented in D_4_ receptor deficient mice. In the retina, clock-dependent dopamine release occurs from the unique population of cells in the inner nuclear layer that are either amacrine or interplexiform neurons [19], which appear to harbor circadian clocks [8]. In addition, the expression of *Drd4*, which encodes the D_4_ receptor, is also circadian in the retina [59]. Thus, circadian/dopaminergic control of *Cpt-1α* may involve both rhythmic dopamine release from amacrine/interplexiform cells and rhythmic expression of its receptor on photoreceptor cells. However, dopaminergic control of *Cpt-1α* appears not to involve modulation of retinal clock function. This follows from the present observation that the daily profile of the clock-controlled gene *Dbp* is unaltered in *Drd*_*4*_^*-/-*^ mice.

The transport of long-chain fatty acids into the mitochondrial matrix is catalyzed by Cpt-1α but that of medium-chain fatty acids occurs through diffusion. Therefore, circadian and dopaminergic regulation of *Cpt-1α* (this study) may account for the control of long-chain FAO but not of medium-chain FAO. The regulatory role of Cpt-1α in long-chain FAO is taken by Acadm—the first enzyme in the ß-oxidation pathway in strict sense—in medium-chain FAO. Like *Cpt-1α*, *Acadm* was presently observed to be under circadian and dopaminergic control and to cycle in PRCs. Therefore, similar regulation of *Cpt-1α* and *Acadm* through retinal clocks and dopamine may ensure consistent adaptation of long-chain and medium-chain FAO and in this way sufficient energy supply to comply with energy demands.

FAO seems to be of physiological importance for retina and PRCs. This is evident from the observation that inherited deficiencies of different enzymes of the mitochondrial FAO pathway and the subsequent impairment of FAO is associated with progressive retinopathy resulting in vision loss [22]. Furthermore, impairment of lipid metabolism [60] and especially of FAO [22, 61] are hallmarks in the pathogenesis of diabetic retinopathy, one of the most common causes of blindness in Europe and USA [62]. The data of the present study extend previous knowledge of an altered daily rhythm of lipid metabolism in the diabetic retina [63] by showing that dysregulation of *Cpt-1α* and *Acadm* might contribute to the impairment of FAO in diabetic retinopathy. They also suggest that dysregulation of Cpt-1 is an overall hallmark of Type II diabetes not only in peripheral tissues (Cpt-1: [64]; Cpt-1β: [65, 66]) but also in retina as an area of the brain/CNS (this study).

In conclusion the data of the present study provide a concept in which circadian and dopamine-dependent regulation of key enzymes of FAO contribute to adaptation of energy metabolism in retina and PRCs and that the disturbance of this regulation might contribute to the pathogenesis of diabetic retinopathy. This suggests that clock-dependent regulation of energy metabolism and its impairment in type II diabetes is evident not only in peripheral organs but also in brain. Finally, it should be mentioned that all mouse models used in this study possess intact PRCs (*rd*^++^) and—with the exception of the db/db mouse as a model for diabetic retinopathy (genetic background: C57BL/6Jb)—were melatonin-proficient (genetic background: C3H/He). Thus the results obtained should mirror general mammalian retinal physiology as close as possible.
